# Supplementary material for: A qnr-plasmid allows aminoglycosides to induce SOS in Escherichia coli
Source: eLife. 2022 Jan 17;11:e69511. doi: 10.7554/eLife.69511 (PMC8789287; doi:10.7554/eLife.69511)
Supplement: Supplementary file 1. [file elife-69511-supp1.docx]

**Supplementary file 1. Genetic background of *qnrD* positive enterobacterial isolates.**

| **Accession number** | **Strains and *qnrD-*plasmids name** | **Size (bp)** | **ORF3** | **ORF4** | **PMQR*** |
| --- | --- | --- | --- | --- | --- |
| JQ776501 | *Escherichia coli* pCGP246 | 4270 |  |  |  |
| JQ776502 | *Escherichia coli* pCGP169 | 4270 |  |  | *qnrS* |
| JQ776504 | *Escherichia coli* pCGB40 | 4269 |  |  |  |
| JQ776506 | *Escherichia coli* pCGS13 | 2687 | + | + | *aac(6’)-lb-cr* |
| JQ776505 | *Citrobacter freundii* pCGF41 | 4268 |  |  |  |
| CP057825 | *Citrobacter freundii* pRHB14-C12 | 2683 | + | + |  |
| JQ776509 | *Klebsiella pneumoniae* pCGH25 | 4270 |  |  | *aac(6’)-lb-cr* |
| FJ228229 | *Salmonella enterica* serovar Bovismorbificans p2007057 | 4270 |  |  |  |
| KJ158441 | *Salmonella enterica* serovar Hadar pQnrD2 | 4268 |  |  |  |
| MK191843 | *Salmonella enterica* serovar Heidelberg | 2683 | + | + |  |
| HF913789 | *Ochrobactrum anthropi* pOA8911 | 2683 | + | + |  |
| HF913788 | *Ochrobactrum anthropi* pOA8917 | 2683 | + | + |  |
| HF679278 | *Octhrobactrum anthropi* pOA8912 | 2683 | + | + |  |
| JX982605 | *Proteus mirabilis* pCGP180 | 4270 |  |  | *aac(6’)-lb-cr* |
| JQ776503 | *Proteus mirabilis* pCGP248 | 2683 | + | + |  |
| JQ776507 | *Proteus mirabilis* pCGS49 | 2683 | + | + | *aac(6’)-lb-cr* |
| JQ776508 | *Proteus mirabilis* pCGH15 | 2683 | + | + |  |
| JX982606 | *Proteus mirabilis* pCGH40 | 4270 |  |  |  |
| KF364953 | *Proteus mirabilis* pRS12-11 | 2683 | + | + |  |
| KF364955 | *Proteus mirabilis* pRS12-104 | 2683 | + | + |  |
| KF364956 | *Proteus mirabilis* pRS12-189 | 2656 | + | + |  |
| KF364957 | *Proteus mirabilis* pRS12-304 | 2658 | + | + |  |
| KJ190020 | *Proteus mirabilis* pM510 | 2683 | + | + |  |
| KF498971 | *Proteus mirabilis* pEAD1-2 | 2669 | + | + |  |
| KP330456 | *Proteus mirabilis* p1042 | 2682 | + | + |  |
| KP313759 | *Proteus mirabilis* pPmZXF | 2683 | + | + |  |
| MF062089 | *Proteus mirabilis* p33184 | 2657 | + | + |  |
| MF062090 | *Proteus mirabilis* p36854 | 2669 | + | + |  |
| MF062091 | *Proteus mirabilis* p39190 | 2669 | + | + |  |
| MF062094 | *Proteus mirabilis* p39224 | 2683 | + | + |  |
| JN183060 | *Proteus mirabilis* pT80 | 2687 | + | + |  |
| CP045539 | *Proteus mirabilis* plB COL3M | 2655 | + | + |  |
| MW248467 | *Proteus mirabilis* pPM1035 | 2683 | + | + |  |
| CP047354 | *Proteus mirabilis* pZA25 | 2683 | + | + |  |
| CP047351 | *Proteus mirabilis* pZN2-*qnrD* | 2683 | + | + |  |
| CP047343 | *Proteus mirabilis* pZF1-*qnrD* | 2683 | + | + |  |
| JX514065 | *Proteus sp.* p3M-2A | 2656 | + | + |  |
| CP047641 | *Proteus sp.* pZN5-*qnrD* | 2683 | + | + |  |
| KF364954 | *Proteus vulgaris* pRS12-78 | 4286 |  |  |  |
| KF498970 | *Proteus vulgaris* pEAD1-1 | 2683 | + | + |  |
| MF062093 | *Proteus vulgaris* p36852 | 2683 | + | + |  |
| CP047348 | *Proteus vulgaris* pZN3-*qnrD* | 2683 | + | + |  |
| MF062092 | *Proteus penneri* p22499 | 2683 | + | + |  |
| CP045011 | *Proteus cibarius* pZF2-*qnrD* | 2683 | + | + |  |
| CP047351 | *Proteus cibarius* pZN2-*qnrD* | 2683 | + | + |  |
| CP047343 | *Proteus cibarius* pZF1-*qnrD* | 2683 | + | + |  |
| HQ834472 | *Providencia rettgeri* pDIJ09-518a | 2683 | + | + |  |
| HQ834473 | *Providencia rettgeri* pGHS09-09a | 2683 | + | + |  |
| MH085193 | *Providencia rettgeri* pAB213 | 2683 | + | + |  |
| MH085194 | *Providencia alcafaciens* pBT169 | 2683 | + | + |  |
| JQ776510 | *Morganella morgani* pCGH69 | 2683 | + | + |  |
| JN183061 | *Morganella morganii* p831 | 2684 | + | + |  |
| KU160530 | *Morganella morganii* pSE10MM | 2662 | + | + |  |
| KF813021 | *Morganella morganii* pM60 | 2683 | + | + |  |

*PMQR other than *qnrD* harboured by the *qnrD*-strain.
