# Supplementary material for: A qnr-plasmid allows aminoglycosides to induce SOS in Escherichia coli
Source: eLife. 2022 Jan 17;11:e69511. doi: 10.7554/eLife.69511 (PMC8789287; doi:10.7554/eLife.69511)
Supplement: Supplementary file 2. [file elife-69511-supp2.docx]

**Supplementary file 2. Minimum-inhibitory concentrations for antibiotics used as SOS-response inducers.**

| **Strains name** | **Tobramycin (µg/L)** | **Gentamicin (µg/mL)** | **Ciprofloxacin (µg/mL)** |
| --- | --- | --- | --- |
| *E. coli* MG1656 | 0.125 | 0.125 | 0.004 |
| *E. coli* MG1656/pDIJ09-518a | 0.125 | 0.125 | 0.094 |
