# Supplementary material for: A qnr-plasmid allows aminoglycosides to induce SOS in Escherichia coli
Source: eLife. 2022 Jan 17;11:e69511. doi: 10.7554/eLife.69511 (PMC8789287; doi:10.7554/eLife.69511)
Supplement: Supplementary file 3. [file elife-69511-supp3.docx]

**Supplementary file 3. Strains and plasmids**

| **Strains** | **Genotype/description** | **References/Sources** |
| --- | --- | --- |
| ***Escherichia coli*** | | |
| MG1655 | K-12 F- lambda- *ilvG*- *rfb*-50 *rph*-1 | (Blattner et al., 1997) |
| MG1655/pDIJ09-518a | MG1655 carrying pDIJ09-518a | This study |
| MG1656 / hereafter called WT | Δ*lacI-lacZ* derivative of MG1655 | Mazel Lab |
| WT/pDIJ09-518a | WT carrying pDIJ09-518a | This study |
| Δ*recA* | Δ*recA*::*tet* | Mazel Lab |
| Δ*recA*/pDIJ09-518a | Δ*recA* carrying pDIJ09-518a | This study |
| DM49 (hereafter called lexAind) | *thr-1, araC14, leuB6, Δ(gpt-proA), lacY1, tsx-33, qsr'-0, glnV44, galK2, LAM-, Rac-0, hisG4, rfbC1, mgl-51,rpsL31, kdgK51, xylA5, mtl-1, argE3, thi-1, lexA3(Ind-)*, TET^R^ | (Mount et al., 1972) |
| *lexA3ind*/pDIJ09-518a | *lexA3(Ind-)* carrying pDIJ09-518a | This study |
| Δ*recB* | MG1655 *recB*::*tet* carrying pG644 | Mazel Lab |
| Δ*recB*/pDIJ09-518a | Δ*recB* carrying pDIJ09-518a | This study |
| Δ*recF* | MG1655 *recF*::*tet* carrying pG644 | Mazel Lab |
| Δ*recF*/pDIJ09-518a | Δ*recF* carrying pDIJ09-518a | This study |
|  |  |  |
| WT/pDIJ09-518aΔ*qnrD* | WT carrying pDIJ09-518a deleted for *qnrD* gene | This study |
| WT/pDIJ09-518aΔORF3 | WT carrying pDIJ09-518a deleted for ORF3 | This study |
| WT/pDIJ09-518aΔORF4 | WT carrying pDIJ09-518a deleted for ORF4 | This study |
| WT/pDIJ09-518a ΔORF3/pORF3 | WT/pDIJ09-518a Δ*ORF3*, complemented with pTOPO::ORF3 | This study |
| WT/pDIJ09-518a ΔORF4/pORF4 | WT/pDIJ09-518a Δ*ORF4*, complemented with pTOPO::ORF4 | This study |
| WT/pDIJ09-518aΔORF3 ΔORF4 | WTcarrying pDIJ09-518a deleted for ORF3 and ORF4*,* CIP^R^ | This study |
| WT::*qnrD* | WT containing chromosomal *qnrD* and its own Pr | This study |
| WT::pDIJ09-518a | WT containing chromosomal native plasmid | This study |
| WT::*qnrD*/pDIJ09-518aΔ*qnrD* | WT containing chromosomal *qnrD* and carrying the deleted *qnrD* plasmid | This study |
| WT/pDIJ09-518a/Pø | WT carrying pDIJ09-518a and pCR2.1 TOPO® TA (empty vector) | This study |
| WT/pDIJ09-518a/pMutT | WT carrying pDIJ09-518a and the vector over-expressing MutT protein | This study |
| WT/pDIJ09-518a/pHmp | WT carrying pDIJ09-518a and the vector over-expressing Hmp protein | This study |
|  |  |  |
| JW2536 (here after called Δ*hmp)* | F-,Δ(*araD-araB*)567, Δ*lacZ*4787(::*rrnB*-3), λ-, Δ*hmp*-726::*kan*, *rph*-1, Δ(*rhaD*-*rhaB*)568, *hsdR*514 | (Baba et al., 2006) |
| Δ*hmp*/pDIJ09-518a | Δ*hmp* carrying pDIJ09-518a | This study |
| Δ*hmp*/pDIJ09-518a/pØ | Δ*hmp* carrying pDIJ09-518a and pCR2.1 TOPO® TA (empty vector) | This study |
| Δ*hmp*/pDIJ09-518a/pHmp | Δ*hmp* carrying pDIJ09-518a and plasmid over-expressing Hmp protein | This study |
| *P. rettgeri*/pDIJ09-518a | *Providencia rettgeri* isolate containing pDIJ09-518a (GenBank accession number HQ834472.1) | (Guillard et al., 2012) |
| TOP10 | Transformation strain | Invitrogen |
| ***Plasmids*** |  |  |
| pDIJ09-518a | Study plasmid | (Guillard et al., 2012) |
| pDIJ09-518a LexA-box* | pDIJ09-518a with *qnrD* constituvely expressed (*qnrD* modified LexA-box) | This study |
| pDIJ09-518aΔ*qnrD* | pDIJ09-518a deleted for *qnrD*, CIP^R^ | This study |
| pDIJ09-518aΔORF3 | pDIJ09-518a deleted for ORF3, CIP^R^ | This study |
| pDIJ09-518aΔORF4 | pDIJ09-518a deleted for ORF4, CIP^R^ | This study |
| pDIJ09-518aΔORF3ΔORF4 | pDIJ09-518a deleted for ORF3 *and* ORF4, CIP^R^ | This study |
| pØ | pCR2.1®-TOPO® TA, KAN^R^ | Thermo Fisher |
| pRecA | pTOPO::RecA, KAN^R^ | This study |
| pORF3 | pTOPO::ORF3, KAN^R^ | This study |
| pORF4 | pTOPO::ORF4, KAN^R^ | This study |
| pMutT | pTOPO::MutT, KAN^R^ | This study |
| pHmp | pTOPO::Hmp, KAN^R^ | This study |
| pG644 | pTOPO::Pr*recN-gfp* | Mazel Lab |
