# Supplementary material for: A qnr-plasmid allows aminoglycosides to induce SOS in Escherichia coli
Source: eLife. 2022 Jan 17;11:e69511. doi: 10.7554/eLife.69511 (PMC8789287; doi:10.7554/eLife.69511)
Supplement: Supplementary file 4. [file elife-69511-supp4.docx]

**Supplementary file 4. Primers used for this study.**

| **Primers** | **Sequence (5’-3’)** |  |
| --- | --- | --- |
| TG01 | GGAGCTGATTTTCGAGGG | To check *qnrD* by sequencing |
| TG02 | AGAAAAATTAGCGTAACTAAGATTTGTC | To check *qnrD* by sequencing |
| LC3 | ATGACGTGGCGATTCAAAA | To amplify *dxs* |
| LC4 | AGCCGGTATAGAGCATCTGG | To amplify *dxs* |
| AB01 | GTTGTCTATCGCGAAGATCAG | To amplify *sfiA* |
| AB02 | GAGCTGGCTAATCTGCATTAC | To amplify *sfiA* |
| AB03 | GCACACCAGCTTCCAGATCA | To amplify *hmp* |
| AB04 | CCTTCCCAACCACCGGCTTT | To amplify *hmp* |
| AB05 | GGTTGTTCAAATTAATGTACAATGATT | To amplify *qnrD* and its own promoter for insertion |
| AB06 | TTTTATCGGTGAACAATAACACCTAA | To amplify *qnrD* and its own promoter for insertion |
| AB07 | CAGTTAAGCCGAAGTATTGA | To amplify native pDIJ09-518a for insertion |
| AB08 | AATTCCCATCGCCTGATAA | To amplify native pDIJ09-518a for insertion |
| AB09 | GCGACGCTTGTTCCTGCG | *cynX* For primer for *qnrD* insertion |
| AB10 | GTACATTAATTTGAACAACCTCTACAT | *cynX-qnrD*  Rev primer |
| AB11 | GAACTTCCTCGCTGAACTTGTA | *qnrD- lacA* For primer |
| AB12 | GAATCGTCAGTTTAATTATAAAAATTGC | *lacA* Rev primer for *qnrD* insertion |
| AB13 | GACAACAACCACTCCGGTTC | *cynX* For primer for plasmid insertion |
| AB14 | CGGCTTAACTGTCTACATTAGC | *cynX*-pDIJ09-518a Rev primer |
| AB15 | GATGGGAATTTCGCTGAACTTG | pDIJ09-518a-*lacA* For primer |
| AB16 | GTATGAGTTTAATCACTCGCATC | *lacA* Rev primer for plasmid insertion |
| AB17 | CTTTCAGCGTAGATTTGGG | To check plasmid insertion by sequencing |
| AB18 | GTTCGCACTTTTCTAATATGACT | To check plasmid insertion by sequencing |
| TG03 | TGTTGAAGTAAAAGGCGCAGG | To amplify *mutT* and its own promoter |
| TG04 | GGCCATTTTGCTCCAAACGT | To amplify *mutT* and its own promoter |
| TG05 | CATCCGCATCTCCTGACTCA | To amplify *hmp* and its own promoter |
| TG06 | GCGCAAACCGGCAAAATCG | To amplify *hmp* and its own promoter |
| TG07 | GTAAAACGACGGCCAGT | To check insert cloned in pTOPO by sequencing |
| TG08 | CAGGAAACAGCTATGAC | To check insert cloned in pTOPO by sequencing |
| AB19 | GATGCAAGGCGAATTAATTTAGATG | To allow *qnrD* deletion |
| AB20 | CTACACCTGGTTATTTATACAGTG | To allow *qnrD* deletion |
| AB21 | TACTGTCTCCGTTCACACATGATCGGAGGGTGTCTCCGTTAGGTTTAC | To allow ORF3 deletion |
| AB22 | GAGACACCCTCCGATCATGTGTGAACGGAG | To allow ORF3 deletion |
| AB23 | GGAGCTTGGTAAACCTCATAAA | To amplify ORF3 and its own promoter |
| AB24 | TAGGTAAACCTAACGTCAGCG | To amplify ORF3 and its own promoter |
| AB25 | TTGCACCCCATGATACTTTCAGTATCCTTTCAGCGATTTC | To allow ORF4 deletion |
| AB26 | GATACTGAAAGTATCATGGGGTGCAA | To allow ORF4 deletion |
| AB27 | CCCGTTATTGCACCCAATTTA | To amplify ORF4 and its own promoter |
| AB28 | CTGACAAGTAAAACGATGCGA | To amplify ORF4 and its own promoter |
| AB29 | TACTGTCTCCGTTCACACATGATCGGAGGGTGTCTCCGTTAGGTTTAC | To allow ORF3 and ORF4 deletion |
| AB30 | GATACTGAAAGTATCATGGGGTGCAA | To allow ORF3 and ORF4 deletion |
| AB31 | ACTGCGTTCGTCTGACCAC | To check pDIJ09-518a plasmid carriage |
| AB32 | TGCGGGCTTCTGAGTGCG | To check pDIJ09-518a plasmid carriage |
| AB33 | GAACCCGTCGTGGTGGAAAT | To amplify *recA* and its own promoter |
| AB34 | TAATCGGTGCCGCGAGTTTA | To amplify *recA* and its own promoter |
| AB35 | TTGCAAGCTATAAATAACCAGGTGT | To modify the LexA-box in *qnrD_*For |
| AB36 | AAAGACAAGATGAAGATGTCGCTTCGC | To modify the LexA-box in *qnrD*_Rev |
| AB37 | ACTGGCAAATGCCCGTTCC | To amplify *katG* |
| AB38 | ACGCGCTTTATCGAGGCTTA | To amplify *katG* |
